# Clinical utility and limitations of transcranial Doppler in evaluating pediatric brain death or death by neurologic criteria: a 10-year retrospective study

**DOI:** 10.3389/fped.2026.1931129

**Published:** 2026-10-20

**Authors:** Na Tan, Yan Li, Ningning Xiao, Quan Wang, Jie Wu, Suyun Qian

**Affiliations:** 1 Pediatric Intensive Care Unit, Beijing Children’s Hospital, Capital Medical University, National Center for Children’s Health, Beijing, China; 2 Department of Emergency Medicine, Beijing Children’s Hospital, Capital Medical University, National Center for Children’s Health, Beijing, China

**Keywords:** brain death, cerebral circulatory arrest, children, death by neurologic criteria, transcranial Doppler

## Abstract

**Objective:**

To evaluate the performance of transcranial Doppler (TCD) in pediatric brain death or death by neurologic criteria (BD/DNC) and factors contributing to discordance between TCD and clinical examination.

**Methods:**

We retrospectively analyzed 512 children admitted to the pediatric intensive care unit between 2016 and 2025 who underwent TCD when the Glasgow Coma Scale score was 3 T. Patients were classified as BD/DNC (*n* = 47), non-BD/DNC (*n* = 266), or incomplete determination (*n* = 199) according to the status of BD/DNC determination. TCD findings and cases of TCD-clinical discordance were analyzed.

**Results:**

TCD findings consistent with BD/DNC were observed in 80.9% (38/47) of the BD/DNC group, none of the non-BD/DNC group, and 58.8% (117/199) of the incomplete determination group. Among 9 patients with clinical examination consistent with BD/DNC but TCD findings inconsistent with BD/DNC, intracranial arterial flow remained detectable. Factors potentially contributing to the discordance included disruption of the closed intracranial system (ventricular drainage, patent anterior fontanelle, or multiple skull fractures; *n* = 4), venoarterial extracorporeal membrane oxygenation support (*n* = 1), cerebrovascular malformations (*n* = 1), and delayed TCD examination (*n* = 1).

**Conclusions:**

TCD findings consistent with BD/DNC strongly support the diagnosis of BD/DNC. Given its noninvasive and bedside applicability, TCD has the potential to be a valuable ancillary tool in pediatric BD/DNC evaluation. However, intracranial arterial flow can still be detected in a subset of children with clinically confirmed BD/DNC, underscoring the importance of considering potential interfering factors when TCD is used as a confirmatory test for pediatric BD/DNC.

## 1 Introduction

Brain death (BD) or death by neurologic criteria (DNC) is defined as the complete and permanent loss of brain function (1). Accurate and timely determination of BD/DNC is essential for end-of-life decision-making. The diagnosis of BD/DNC is based on clinical examination, while ancillary tests are used to support the clinical conclusion, particularly when clinical examination is restricted or inconclusive. However, in some countries, including China, ancillary testing is not optional but constitutes a mandatory component of the BD/DNC determination.

Transcranial Doppler (TCD) identifies cerebral circulatory arrest by assessing intracranial arterial spectral waveforms and offers several advantages, including noninvasiveness, repeatability, bedside applicability, relatively low cost, and insensitivity to sedative agents. Its application in adult BD/DNC determination is well established (2, 3). However, the use of TCD in pediatric BD/DNC determination remains controversial. Anatomical characteristics in infants and young children, such as open fontanelles and cranial sutures, can partially buffer increases in intracranial pressure (ICP), potentially reducing the sensitivity of TCD (4). In addition, the evidence on TCD diagnostic performance in children is limited, with most studies involving small sample sizes and substantial methodological heterogeneity, which restricts the robustness and generalizability of the findings (5–8). In this context, recent guidelines from the United States, Canada, Australia, and New Zealand do not recommend TCD as a confirmatory test for pediatric BD/DNC determination (9–11).

Therefore, we conducted a retrospective study of children with severe brain injury to evaluate the performance of TCD in BD/DNC evaluation and, in particular, to characterize cases in which TCD findings were discordant with clinical examination.

## 2 Materials and methods

### 2.1 Study design and participants

This study was conducted in accordance with the Declaration of Helsinki and was approved by the Medical Ethics Committee of Beijing Children's Hospital, Capital Medical University (Approval Code: 2026-E-056-R; Approval Date: 13 April 2026). The requirement for informed consent was waived owing to the retrospective nature of the study.

We retrospectively analyzed the electronic medical records of patients admitted to the Pediatric Intensive Care Unit (PICU) of Beijing Children's Hospital, Capital Medical University, between 2016 and 2025, who had a Glasgow Coma Scale (GCS) score of 3 T (eye opening = 1, motor response = 1, and verbal response not assessable due to endotracheal intubation) unexplained by sedation and underwent TCD examination on the same day. Patients were excluded if TCD waveforms consistent with BD/DNC were identified in accessible intracranial arteries, while evaluation of the anterior and posterior circulations could not be fully achieved.

According to the status of BD/DNC determination, participants were classified into three groups: (a) the BD/DNC group, comprising patients in whom BD/DNC was confirmed according to the Chinese pediatric criteria (12, 13); (b) the non-BD/DNC group, comprising patients in whom BD/DNC was excluded on the basis of preserved brain function, such as retained brainstem reflexes or spontaneous respiration, and therefore no BD/DNC evaluation was initiated; and (c) the incomplete determination group, comprising patients in whom BD/DNC was not excluded on clinical grounds but confirmation could not be achieved because the determination process was not initiated or completed.

All patients in the BD/DNC group fulfilled clinical criteria for BD/DNC in the absence of confounders. Accordingly, the final clinical determination was used as the reference standard for evaluating TCD performance. Within the BD/DNC group, patients were further classified into TCD-consistent and TCD-inconsistent subgroups based on whether TCD findings were consistent with BD/DNC criteria.

Nine of the children included in the BD/DNC group of the present study had also been reported in our previous publications, including two children reported in (14), five in (15), and one each in (16) and (17). These publications addressed practical aspects and challenges of pediatric BD/DNC determination, as well as computed tomography (CT) angiography and perfusion findings in children with BD/DNC. Of these nine children, eight were classified into the TCD-consistent subgroup (14–16) and one into the TCD-inconsistent subgroup (17).

### 2.2 TCD examination

In our PICU, TCD has been routinely incorporated into the neurocritical care protocol for children with severe disorders of consciousness (DoC), defined as a GCS score ≤ 8 or the need for endotracheal intubation due to impaired consciousness, to assess cerebral hemodynamics and indirectly monitor ICP trends (18). TCD examinations were performed using a Sonara system (Cardinal Health 209 Inc., Madison, WI, USA) with a 2.0-MHz probe before April 2023 and an EMS-9F system (Delica Medical, Shenzhen, China) with a 1.6-MHz probe thereafter. Sweep speed was set at 4–8 s, sample volume (gate) at 4–18 mm, and a low wall filter (≤ 50 Hz) was applied. Routine insonation was performed through the temporal, suboccipital, and submandibular windows to assess the middle cerebral artery (MCA), terminal internal carotid artery (ICA), anterior cerebral artery, posterior cerebral artery, basilar artery (BA), intracranial vertebral artery (VA), and extracranial ICA. When the temporal window was inadequate or inaccessible, the carotid siphon (CS) was assessed through the orbital window. All examinations were performed by two full-time technicians certified in TCD evaluation for pediatric BD/DNC determination in China (19), who originally interpreted the TCD findings at the time of examination. During data analysis, the stored TCD waveforms from all included patients were independently re-interpreted by a third study investigator who was also certified in TCD evaluation and blinded to all other clinical and ancillary test information. The re-interpreted findings were fully concordant with the original interpretations regarding whether the TCD findings were consistent with BD/DNC.

### 2.3 Determination of BD/DNC

BD/DNC determination was performed in accordance with the Chinese pediatric criteria (2014 or 2019 version of the *Criteria and Practical Guidance for Determination of Brain Death in Children*), depending on the date of evaluation (12, 13). Before initiating a BD/DNC evaluation, the etiology of devastating brain injury was established, and confounders were excluded. The declaration of BD/DNC required two sets of examinations that were both consistent with BD/DNC, each comprising a clinical examination and confirmatory tests. The two sets of examinations were performed by at least two clinicians certified in pediatric BD/DNC determination in China, with an age-dependent observation period between them: a minimum interval of 24 h for infants aged 29 days to < 1 year, and 12 h for children aged 1–18 years.

The components of the BD/DNC clinical examination included: (a) coma, confirmed by the absence of any facial muscle movement in response to deep pressure over the bilateral supraorbital notches or pinprick stimulation to the face, with a GCS score of 3 T; (b) brainstem areflexia, including pupillary, cough, corneal, oculocephalic, and oculovestibular reflexes; (c) apnea, confirmed by no respiratory movement, no patient-triggered ventilator breaths, and a positive apnea test result. The principal difference between the 2014 and 2019 versions concerned the target arterial carbon dioxide tension (PaCO_2_) for the apnea test. The 2014 version indicated that the PaCO_2_ threshold for determination of BD/DNC during apnea testing was ≥ 60 mm Hg or 20 mm Hg over baseline (12), whereas the 2019 version required both thresholds to be met simultaneously (13). The time at which clinical examination met BD/DNC criteria was defined as the moment a positive apnea test was completed.

In China, TCD, electroencephalography (EEG), and short-latency somatosensory evoked potentials (SLSEP) were permissible confirmatory tests. At least two of these tests must be consistent with BD/DNC to declare BD/DNC; this number increases to three when any brainstem reflex cannot be tested. TCD findings were considered consistent with BD/DNC when the anterior and posterior circulations bilaterally demonstrated reverberating flow, systolic spikes, or loss of previously detectable intracranial flow signals on serial examinations during the same hospitalization. The MCA was the preferred vessel for anterior circulation assessment (with the intracranial ICA as an alternative), and the BA was preferred for posterior circulation assessment (with the intracranial VA as an alternative). Reverberating flow was defined as forward systolic flow accompanied by reverse diastolic flow within a single cardiac cycle, with a direction of flow index < 0.8, calculated as 1−(reverse velocity/forward velocity). Systolic spikes were defined as isolated systolic flow signals with a duration < 200 ms and a peak systolic velocity < 50 cm/s. EEG findings were considered consistent with BD/DNC when prolonged recordings demonstrated electrocerebral inactivity (electrical activity ≤ 2 *μ*V). The minimum recording duration was 30 min for children older than 2 months and 60 min for infants 2 months of age or younger. SLSEP findings were considered consistent with BD/DNC when electrical stimulation of the bilateral median nerves elicited brachial plexus (N9) potentials, with preservation or absence of cervical cord (N13) potentials, and absence of brainstem (P14 and N18) and cortical (N20) potentials.

### 2.4 Statistical analysis

Statistical analyses were performed using R version 4.3.3 (R Foundation for Statistical Computing, Vienna, Austria). Normality was assessed using the Shapiro–Wilk test. As all continuous variables were nonnormally distributed, they were presented as medians and interquartile ranges (IQRs). Categorical variables were expressed as counts and percentages. Comparisons between TCD-consistent and TCD-inconsistent subgroups within the BD/DNC group were performed using the Mann–Whitney *U* test for continuous variables, and the chi-square (*χ^2^*) test or Fisher's exact test for categorical variables. A two-sided *P* value of < 0.05 was considered statistically significant.

## 3 Results

### 3.1 Baseline characteristics

During the 10-year study period, 516 patients met the inclusion criteria. Four were excluded because severe craniofacial trauma (*n* = 1) or occipital surgical wounds (*n* = 3) precluded complete assessment of the anterior and posterior circulations, despite TCD waveforms consistent with BD/DNC being identified in accessible intracranial arteries. Ultimately, 512 patients were included in the analysis. The median age was 81 months (IQR: 33–130 months, range: 1 month–17.6 years), and 304 (59.4%) were male. The most common etiologies of brain injury were infection-related encephalopathy (21.9%), hypoxic-ischemic brain injury (20.9%), and traumatic brain injury (13.7%). The median number of TCD examinations was 2 (IQR: 1–3). Most patients (94.5%, 484/512) underwent their first TCD examination within 3 days of PICU admission, and the median interval from the onset of severe DoC to the first TCD examination was 1 day (IQR: 1–3 days). Overall, 47 patients (9.2%) were classified into the BD/DNC group, 266 (52.0%) into the non-BD/DNC group, and 199 (38.8%) into the incomplete determination group. Baseline characteristics of the three groups are presented in Table 1.

**Table 1:** Baseline characteristics of participants.

Variable	Total (*n* = 512)	BD/DNC (*n* = 47)	Non-BD/DNC (*n* = 266)	Incomplete determination (*n* = 199)
Male sex	304 (59.4)	26 (55.3)	162 (60.9)	116 (58.3)
Age, months	81 (33, 130)	78 (39, 115)	88 (43, 137)	63 (29, 124)
Age groups				
1 to < 12 months	39 (7.6)	2 (4.3)	20 (7.5)	17 (8.5)
12 to < 24 months	45 (8.8)	4 (8.5)	22 (8.3)	19 (9.6)
24 months to < 18 years	428 (83.6)	41 (87.2)	224 (84.2)	163 (81.9)
Etiology of brain injury				
Infection-related encephalopathy	112 (21.9)	6 (12.8)	66 (24.8)	40 (20.1)
Hypoxic-ischemic brain injury	107 (20.9)	17 (36.2)	48 (18.1)	42 (21.1)
Traumatic brain injury	70 (13.7)	13 (27.6)	33 (12.4)	24 (12.1)
Cerebrovascular events	48 (9.4)	3 (6.4)	20 (7.5)	25 (12.6)
Intracranial infection	42 (8.2)	4 (8.5)	25 (9.4)	13 (6.5)
Intracranial neoplasm	37 (7.2)	3 (6.4)	17 (6.4)	17 (8.6)
Metabolic or toxic brain injury	36 (7.0)	0 (0.0)	25 (9.4)	11 (5.5)
Others	32 (6.2)	1 (2.1)	20 (7.5)	11 (5.5)
Unknown	28 (5.5)	0 (0.0)	12 (4.5)	16 (8.0)
Number of TCD examinations	2 (1, 3)	4 (3, 5)	2 (1, 3)	2 (1, 3)
Time from severe DoC onset to first TCD, days	1 (1, 3)	1 (1, 3)	1 (1, 3)	1 (1, 3)
PICU length of stay, days	7 (3, 18)	8 (6, 11)	14 (5, 27)	3 (1, 8)
Total hospital length of stay, days	11 (3, 28)	9 (6, 12)	21 (9, 40)	4 (2, 9)
Clinical outcome at discharge				
In-hospital cardiac death	152 (29.7)	23 (48.9)	25 (9.4)	104 (52.3)
Discharged at family's request^a^	188 (36.7)	24 (51.1)	69 (25.9)	95 (47.7)
Improved at discharge	172 (33.6)	0 (0.0)	172 (64.7)	0 (0.0)
Organ or cornea donation	8 (1.6)	4 (8.5)	0 (0.0)	4 (2.0)

Continuous variables are presented as median (interquartile range), and categorical variables as *n* (%). BD, brain death; DNC, death by neurologic criteria; TCD, transcranial Doppler; DoC, disorders of consciousness; PICU, pediatric intensive care unit. ^a^Includes patients discharged from our hospital at the family's request following BD/DNC declaration or in the setting of a critically poor prognosis.

### 3.2 TCD findings

TCD findings consistent with BD/DNC were observed in 80.9% of patients in the BD/DNC group and in none of the patients in the non-BD/DNC group. Within the incomplete determination group, TCD findings consistent with BD/DNC were observed in 52.9% of patients in whom BD/DNC evaluation was not initiated, 66.7% of those in whom apnea testing was not completed, and 100.0% of those in whom repeat examination was not completed (Table 2). Detailed patterns of ancillary testing, including TCD, EEG, and SLSEP, and the corresponding findings across the three groups are summarized in Supplementary Table S1. Among the 43 patients who fulfilled BD/DNC criteria based on clinical examination, EEG, and SLSEP findings, TCD findings were consistent with BD/DNC in 34 patients (79.1%).

**Table 2:** TCD findings in the BD/DNC, non-BD/DNC, and incomplete determination groups.

TCD findings	BD/DNC (*n* = 47)	Non-BD/DNC (*n* = 266)	Incomplete determination^a^		
			Evaluation not initiated (*n* = 157)	Apnea testing not completed (*n* = 24)	Repeat examination not completed (*n* = 18)
Consistent with BD/DNC	38 (80.9)	0 (0.0)	83 (52.9)	16 (66.7)	18 (100.0)^b^
Inconsistent with BD/DNC	9 (19.1)^c^	266 (100.0)	74 (47.1)	8 (33.3)	0 (0.0)

Variables are presented as *n* (%). TCD, transcranial Doppler; BD, brain death; DNC, death by neurologic criteria. ^a^Eight patients showed no flow signals on the initial TCD examination, including seven in whom BD/DNC evaluation was not initiated and one in whom apnea testing was not completed. ^b^Sixteen patients had at least two confirmatory tests consistent with BD/DNC. ^c^One patient showed no flow signals on the initial TCD examination.

### 3.3 Timing of TCD examinations in the BD/DNC group

Among the 47 patients in the BD/DNC group, 44 underwent TCD examination before the initial clinical examination met BD/DNC criteria. Of these, 34 (77.3%) already had TCD findings consistent with BD/DNC at a median of 28.4 h (IQR: 16.0–59.1 h) before this time point. In one patient, a repeat TCD examination performed 5 h after the initial clinical examination met BD/DNC criteria subsequently showed findings consistent with BD/DNC. Four patients had persistent TCD findings inconsistent with BD/DNC on follow-up examinations, and five did not undergo further TCD examinations. The remaining three patients underwent their first TCD examination 2–16 h after the initial clinical examination met BD/DNC criteria, and all demonstrated findings consistent with BD/DNC (Figure 1).

**Figure 1:**
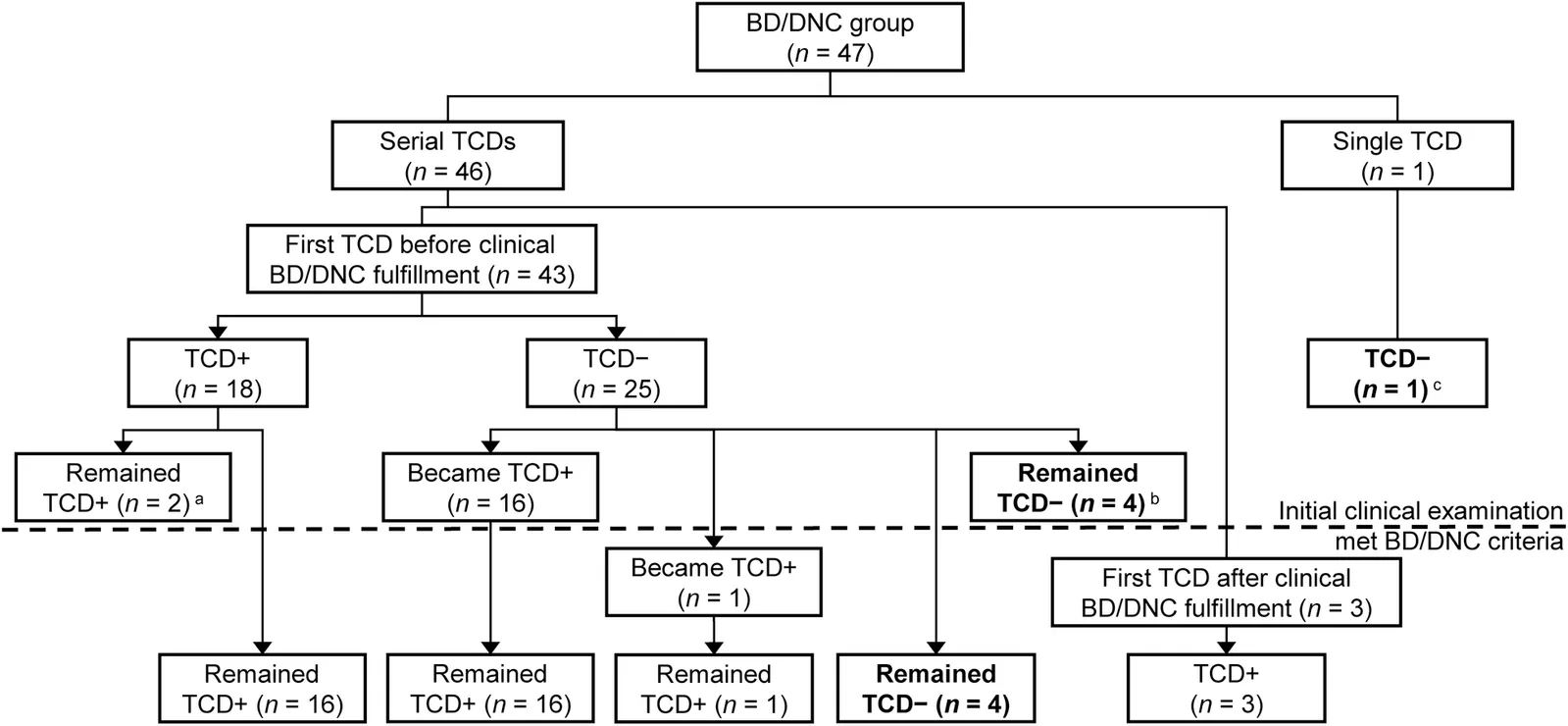
Timing and findings of TCD examinations in the BD/DNC group. BD, brain death; DNC, death by neurologic criteria; TCD, transcranial Doppler. “TCD+” indicates TCD findings consistent with BD/DNC. “TCD−” indicates TCD findings inconsistent with BD/DNC. Bold indicates cases with discordance between TCD and clinical examination. The reference time point is when the initial clinical examination met BD/DNC criteria. ^a^The last TCD examination was performed 5 and 11 h before the reference time point, respectively. ^b^The last TCD examination was performed 3–25 h before the reference time point. ^c^The TCD examination was performed 4 days before the reference time point.

### 3.4 Characteristics of patients with TCD findings inconsistent with BD/DNC in the BD/DNC group

No statistically significant differences were identified between the TCD-consistent and TCD-inconsistent subgroups across demographics, etiology, clinical features, TCD utilization characteristics, or clinical outcomes (all *P* > 0.05). Nevertheless, patients in the TCD-inconsistent subgroup tended to have a higher frequency of ventricular drainage, fewer TCD examinations, longer PICU stay after declaration of BD/DNC, and longer total hospital stay (Table 3).

**Table 3:** Comparison of characteristics between TCD-consistent and TCD-inconsistent subgroups within the BD/DNC group.

Variable	TCD-consistent (*n* = 38)	TCD-inconsistent (*n* = 9)	*P* value
Male sex	21 (55.3)	5 (55.6)	> 0.999
Age, months	78 (38, 109)	87 (44, 122)	0.735
Age group			0.322
1 to < 12 months	1 (2.6)	1 (11.1)	
12 to < 24 months	3 (7.9)	1 (11.1)	
24 months to < 18 years	34 (89.5)	7 (77.8)	
Etiology of brain injury			0.988
Hypoxic-ischemic brain injury	14 (36.8)	3 (33.3)	
Traumatic brain injury	10 (26.3)	3 (33.3)	
Infection-related encephalopathy	5 (13.2)	1 (11.1)	
Cerebrovascular events	2 (5.3)	1 (11.1)	
Intracranial infection	3 (7.9)	1 (11.2)	
Intracranial neoplasm	3 (7.9)	0 (0.00)	
Other	1 (2.6)	0 (0.00)	
Conditions associated with disruption of the closed intracranial system	11 (28.9)^a^	4 (44.4)^b^	0.618
Skull fracture	8 (21.1)	2 (22.2)	> 0.999
Patent fontanelle	2 (5.3)	1 (11.1)	0.480
Decompressive craniectomy	1 (2.6)	0 (0.0)	> 0.999
Ventricular drainage	1 (2.6)	2 (22.2)	0.090
BD/DNC determination criteria			> 0.999
2014 version	11 (28.9)	2 (22.2)	
2019 version	27 (71.1)	7 (77.8)	
TCD system			0.422
Sonara + 2.0-MHz probe	25 (65.8)	4 (44.4)	
EMS-9F + 1.6-MHz probe	13 (34.2)	5 (55.6)	
Time from severe DoC onset to first TCD, days	1 (1, 2)	3 (1, 9)	0.240
Number of TCD examinations	4 (3, 5)	2 (2, 3)	0.062
PICU stay after BD/DNC declaration, days	1 (1, 3)	5 (3, 8)	0.087
PICU length of stay, days	8 (6, 11)	10 (8, 13)	0.272
Total hospital length of stay, days	9 (6, 11)	11 (9, 13)	0.093

Continuous variables are presented as median (interquartile range), and categorical variables as *n* (%). TCD, transcranial Doppler; BD, brain death; DNC, death by neurologic criteria; DoC, disorders of consciousness; PICU, pediatric intensive care unit. ^a^One patient had both skull fracture and ventricular drainage. ^b^One patient had both patent fontanelle and ventricular drainage.

The clinical course, electrophysiological findings, and temporal evolution of TCD flow patterns in the nine TCD-inconsistent subgroup patients are summarized in Table 4. Four patients had disruption of the closed intracranial system, including ventricular drainage, patent anterior fontanelle, or multiple skull fractures. Continuous forward diastolic flow was detected in certain intracranial arteries in these cases (Cases 2–4 and 7, Supplementary Figure S1). One patient with cerebrovascular malformation exhibited asymmetric cerebral perfusion 21 h before the initial clinical examination met BD/DNC criteria, with residual forward flow on the affected side and markedly reduced or absent flow contralaterally (Case 1, Supplementary Figure S2). One patient with hypoxic-ischemic brain injury underwent TCD examination 28 days after the onset of severe DoC and showed only reduced diastolic blood velocity (Case 5, Supplementary Figure S3). One patient supported by venoarterial extracorporeal membrane oxygenation (VA-ECMO) underwent TCD examination that demonstrated a transition from nonpulsatile to pulsatile flow, but forward diastolic flow remained present (Figure 2). In this patient, CT angiography and perfusion imaging performed on day 13 of severe DoC indicated cerebral circulatory arrest (Case 8). No definite contributing factors were identified in the remaining two patients (Cases 6 and 9).

**Table 4:** Temporal evolution of clinical, electrophysiological, and cerebral blood flow findings in the TCD-inconsistent subgroup within the BD/DNC group.

Case	Age/Sex/Primary diagnosis	Clinical exam	EEG	SLSEP	TCD waveforms	Possible explanations for persistent flow	Outcome
1	6 y/F/Left cerebrovascular malformation (AVM highly suspected) with ICH	+ (D5, 6)	+ (D4, 5, 6)	+ (D4, 6)	D2: LMCA and left terminal ICA blood velocity increased (PSV 183–196 cm/s), no other abnormal findings D4: LCS blood velocity decreased (PSV 73 cm/s); RCS and LVA CLBV; RVA no signal	1) Low-resistance shunt pathway due to AVM; 2) Examination performed prior to cerebral circulatory arrest	D6: Discharged on organ support
2	10 y/M/Fungal meningitis	+ (D14, 15)	+ (D14, 15)	+ (D14, 15)	D11–D12: LMCA blood velocity decreased (PSV 33–71 cm/s); RMCA SS or RF; BA SS D14: MCAs blood velocity decreased (PSV 43–64 cm/s); VAs CLBV	Left ventricular external drainage (D4) mitigating ICP elevation	D15: Discharged on organ support
3	11 mon/F/Purulent meningitis, HIBI (cardiac arrest)	+ (D5, 6)	− (D2, 3); + (D5, 7)	+ (D5, 7)	D2–D7: High-flow, low-resistance pattern; LI peaked at 9.5 on D5 and decreased to 4.6 on D6 and 2.1 on D7 during nimodipine infusion (D4–D7)	V-P shunt and patent fontanelle mitigating ICP elevation	D21: Spontaneous cardiac arrest
4	10 y/F/Traumatic brain injury	+ (D12, 28, 30)^a^	+ (D12, 13, 28, 30)	+ (D12, 14, 28, 30)	D4: MCA blood velocity increased (PSV 260 cm/s), LI 3.2, no other abnormal findings D7–D8: LCS SS; RCS CLBV; posterior circulation no signal D11–D12: CSs CLBV; BA RF D13–D14: LCS blood velocity decreased (PSV 31–58 cm/s); RCS no signal; BA CLBV D15–D42: anterior circulation CLBV; posterior circulation SS or no signal	Multiple skull fractures mitigating ICP elevation	D49: Spontaneous cardiac arrest
5	3 y/M/HIBI (asphyxia)	+ (D32, 33)	+ (D28, 31, 33)	+ (D31, 33)	D28: Anterior and posterior circulations diastolic blood velocity decreased	Resolution of cerebral edema reducing ICP	D36: Discharged on organ support
6	1 y 11 mon/M/Acute necrotizing encephalopathy	+ (D14, 15)	− (D8); + (D14, 15)	+ (D14, 15)	D10: LMCA SS; RMCA RF; BA systolic peaks (PSV 26 cm/s, duration 227 ms) D15: LMCA and BA SS; RMCA systolic peaks (PSV 92 cm/s, duration 75 ms) D16: LCS systolic peaks (PSV 98 cm/s, duration 438 ms); RCS and BA SS	No identifiable explanation	D18: Discharged on organ support
7	10 y/M/Traumatic brain injury	+ (D5, 6)	+ (D5, 6)	+ (D5, 6)	D2: LCS and RMCA SS; BA blood velocity decreased (PSV 61 cm/s) D4: LCS SS; RCS CLBV; BA blood velocity decreased (PSV 61 cm/s)	Multiple skull fractures mitigating ICP elevation	D11: Organ donation
8	9 y/M/ Catecholaminergic polymorphic ventricular tachycardia	+ (D8, 9)^b^	+ (D8, 9)	+ (D8, 9)	D2: CSs nonpulsatile CLBV; posterior circulation not assessed (LVEF 24%) D8: LCS and RMCA pulsatile CLBV; BA PSV 49 cm/s, duration 472 ms, near-absent mid-to-late diastolic flow (LVEF 26%)	VA-ECMO (D2–D14; common carotid artery cannulation) providing nonpulsatile flow	D14: Withdrawal of life-sustaining therapy followed by cardiac arrest
9	7 y/F/Traumatic brain injury	+(D6, 6)^c^	+(D6, 6)^c^	+(D6, 6)^c^	D4: Anterior and posterior circulations no signal D6: LCS and RMCA CLBV; BA RF	No identifiable explanation	D14: Discharged on organ support

D1 indicates the day of onset of severe disorder of consciousness. “−” indicates inconsistent with BD/DNC. “+” indicates consistent with BD/DNC. CLBV indicates continuous forward flow with a PSV < 50 cm/s. LI = mean blood velocity of the MCA/mean blood velocity of the ipsilateral extracranial ICA. Pupillary reflex loss and tracheal intubation occurred on day 1 in most patients, with delayed occurrence in Case 1 (D3, D3), Case 2 (D10, D10), and Case 4 (D7, D3). Cases 3, 4, 6, and 9 underwent TCD after the initial clinical examination met BD/DNC criteria. TCD, transcranial Doppler; BD, brain death; DNC, death by neurologic criteria; EEG, electroencephalography; SLSEP, short-latency somatosensory evoked potential; y, year; mon, month; F, female; M, male; AVM, arteriovenous malformation; ICH, intracranial hemorrhage; HIBI, hypoxic-ischemic brain injury; D, day; L, left; R, right; MCA, middle cerebral artery; ICA, internal carotid artery; CS, carotid siphon; VA, vertebral artery; BA, basilar artery; PSV, peak systolic velocity; CLBV, continuous low blood velocity; RF, reverberating flow; SS, systolic spikes; LI, Lindegaard index; ICP, intracranial pressure; V-P ventriculoperitoneal; LVEF, left ventricular ejection fraction; VA-ECMO, venoarterial extracorporeal membrane oxygenation. ^a^The apnea test on D13 showed no spontaneous respiration but was terminated early due to hypotension. ^b^Apnea tests were performed by reducing the ECMO sweep gas flow rate to 0.5 L/min. ^c^Clinical examination, EEG, and SLSEP were each performed twice on D6, separated by a 13-hour interval.

**Figure 2:**
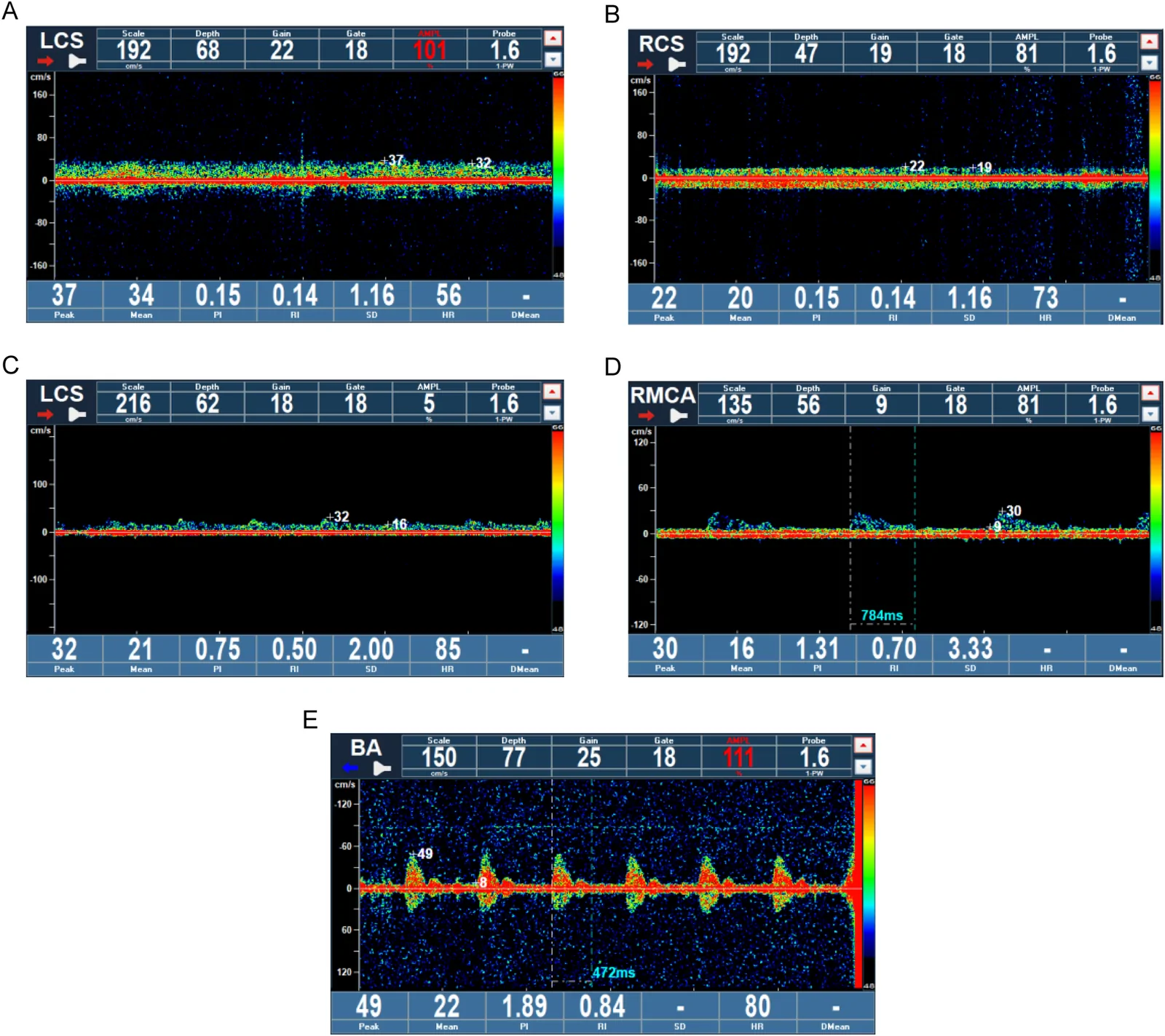
Evolution of TCD spectral waveforms in case 8 during veno-arterial extracorporeal membrane oxygenation support. The flow pattern changed from nonpulsatile (**A**, **B**; day 2 of severe disorders of consciousness) to pulsatile (**C–E**; day 8 of severe disorders of consciousness). AMPL: amplitude; Peak: peak systolic velocity; Mean: mean velocity; Dias: end-diastolic velocity; PI: pulsatility index; SD: systolic-to-diastolic ratio; HR: heart rate; LMCA: left middle cerebral artery; RMCA: right middle cerebral artery.

Neuroimaging evidence of intracranial hypertension, including severe cerebral edema and herniation, was present in 7 of the 9 patients. The two exceptions were Cases 3 and 5. In Case 3, cranial CT on day 1 of severe DoC demonstrated hydrocephalus with periventricular edema, but no follow-up neuroimaging was obtained because guardian consent was declined. In Case 5, brain magnetic resonance imaging at an outside hospital on day 26 of severe DoC demonstrated extensive hypoxic-ischemic brain injury, and no repeat neuroimaging was performed after transfer to our institution.

## 4 Discussion

In this single-center, 10-year cohort of children with severe brain injury, we evaluated the clinical utility and limitations of TCD in pediatric BD/DNC evaluation. No TCD findings consistent with BD/DNC were observed in the non-BD/DNC group, supporting the high specificity of TCD (100%, 266/266). However, only 81% (38/47) of children in the BD/DNC group demonstrated TCD findings consistent with BD/DNC. This proportion is comparable to the sensitivities reported in two recent larger pediatric cohorts [88% [58/66] and 76% [65/85], respectively] (7, 20), indicating that false-negative TCD findings are not uncommon in pediatric BD/DNC evaluation.

In countries such as China, where confirmatory testing is mandatory for BD/DNC determination, discordance between clinical examination and TCD findings may raise concerns among clinicians and families regarding the diagnosis. China has not yet enacted legislation on BD/DNC. In our clinical practice, discontinuation of organ support after BD/DNC determination requires the consent of the patient's family. In our cohort, children with clinically confirmed BD/DNC but TCD findings inconsistent with BD/DNC tended to have prolonged PICU stay after BD/DNC declaration and longer total hospital stay, which may suggest that such discordance complicates clinical decision-making. Identification of factors associated with persistent intracranial arterial flow signals after BD/DNC may therefore facilitate appropriate interpretation of TCD findings, help define the limitations of TCD in pediatric BD/DNC evaluation, and reduce unwarranted doubts regarding the clinical diagnosis.

In the present study, false-negative TCD findings were predominantly characterized by markedly reduced but persistent forward diastolic flow. This pattern may represent an evolving transition toward cerebral circulatory arrest or indicate that residual cerebral perfusion has fallen below the threshold required to sustain brain function. Notably, although Cases 3 and 5 had potential interfering factors, including ventricular drainage and delayed TCD assessment, they exhibited neither substantially reduced intracranial arterial blood velocities nor definitive neuroimaging evidence of intracranial hypertension. Clinicians should exercise caution in such cases. Prolonged observation, repeat TCD examinations, and, when appropriate, additional cerebral blood flow assessments may improve the accuracy and reliability of BD/DNC determination (21, 22).

Consistent with previous reports (4, 5, 7, 23), the most common cause of false-negative TCD findings in this study was disruption of the closed intracranial system. Factors such as ventricular drainage, decompressive craniectomy, patent fontanelle, and multiple skull fractures can mitigate ICP elevation, delaying the emergence of characteristic TCD patterns of cerebral circulatory arrest.

Abnormal cerebrovascular architecture may also interfere with TCD findings. Ergun et al. (24) reported a child fulfilling the clinical criteria for BD/DNC, in whom conventional angiography showed right ICA flow up to the posterior communicating artery with opacification of the right posterior cerebral artery and visualization of an arteriovenous malformation (AVM), which was also seen on VA angiography. No opacification of the circle of Willis or the anterior and middle cerebral arteries was observed in any selective angiograms. This suggests that AVMs may act as low-resistance shunts, maintaining a local pressure gradient that drives blood from the arterial to the venous system despite markedly elevated ICP. Case 1 in our study exhibited a similar pattern: despite electrophysiological evidence of BD/DNC, TCD detected residual forward flow on the affected side, while flow on the contralateral side was markedly reduced or absent. Therefore, in patients with AVM, TCD signals should be interpreted alongside neuroimaging to avoid overestimating cerebral perfusion.

Continuous nonpulsatile flow from VA-ECMO can distort TCD findings. Adult studies indicate that in BD/DNC patients, a left ventricular ejection fraction (LVEF) exceeding 10%–20% or the use of intra-aortic balloon pump support is often associated with preservation of typical cerebral circulatory arrest patterns, whereas severely impaired cardiac function may result in nonpulsatile signals (25–27). Previous reports of VA-ECMO-supported children with BD/DNC have most commonly reported absent intracranial flow signals on TCD (28–32). Case 8 initially showed nonpulsatile flow despite an LVEF > 20%, which may be related to altered cerebral hemodynamics from common carotid artery cannulation in children (33). During subsequent monitoring, BD/DNC was confirmed by clinical and electrophysiological criteria. Although partial restoration of cardiac pulsatility was observed, TCD continued to detect low-velocity forward diastolic flow. This suggests that even with cardiac pulsatility, the ECMO driving pressure may maintain continuous intracranial flow, thereby masking typical reverberating flow or systolic spike patterns. CT angiography and perfusion imaging performed 5 days after the final TCD examination demonstrated cerebral circulatory arrest. However, without contemporaneous TCD data, whether the flow pattern had evolved to one consistent with cerebral circulatory arrest remains uncertain.

TCD detection of cerebral circulatory arrest is time dependent, with a reported rate of 58% within 6 h of clinical BD/DNC diagnosis, increasing to 100% after 24–36 h (34). However, in most patients in our study, TCD evidence of cerebral circulatory arrest was detected before completion of clinical BD/DNC determination. This may stem from the inherent delay in fulfilling strict prerequisites and observation intervals. Furthermore, the Chinese protocol adopts a strategy in which confirmatory tests are performed before apnea testing (12, 13), resulting in earlier TCD assessment within the diagnostic sequence. We also observed residual flow in a patient with a prolonged disease course, in whom BD/DNC was subsequently confirmed (Case 5). A possible explanation is that resolution of cerebral edema and resorption of necrotic tissue during the subacute or chronic stage reduced ICP after the loss of brain function (35–37). However, this hypothesis could not be verified because serial neuroimaging and TCD data were unavailable.

Beyond false negatives from persistent flow, another challenge in the use of TCD for BD/DNC diagnosis is the absence of detectable flow signals. Unlike adults, children's thinner temporal bones and incomplete skull ossification provide superior acoustic windows for intracranial blood flow assessment (38). In this study, only nine patients had absent flow signals on their initial TCD examination. Among them, one was ultimately declared BD/DNC, whereas the remaining eight belonged to the incomplete determination group. Although current guidelines remain cautious in equating signal absence with cerebral circulatory arrest due to potential technical limitations (8), our results suggest that, when confirmed by experienced operators, the absence of signals in both anterior and posterior circulations across multiple windows should raise strong suspicion of BD/DNC.

False-positive TCD findings, where clinical examination is not consistent with BD/DNC but TCD suggests BD/DNC, are rarely reported and typically characterized by reverberating flow associated with hypotension, systemic-to-pulmonary shunts, or aortic regurgitation (6, 39). No false positives were identified in this study, which may be attributable to maintenance of blood pressure at high-normal levels or above to preserve cerebral perfusion, driven by the Cushing response and intensive hemodynamic support. Special consideration is warranted in young infants. During case screening, a 2-month-old term infant with diffuse cerebral edema of unknown etiology was identified but excluded because the patient's lowest GCS score during hospitalization was 5 T (eye opening = 1, motor response = 3, and verbal response not assessable due to endotracheal intubation). The infant showed persistent reverberating flow and absent brainstem reflexes. The discrepancy between these findings and the observed motor responses may have been related to lower cerebral blood flow requirements and neurological immaturity in young infants (40). Alternatively, the observed limb movements may have represented spinal reflex activity.

This study has several limitations. First, the timing and frequency of TCD examinations were not standardized, and some patients did not undergo repeat TCD after fulfilling clinical criteria for BD/DNC, potentially underestimating the proportion of patients who would eventually develop TCD findings consistent with BD/DNC. Second, due to clinical instability or guardian preference, the determination process could not be initiated or completed in some patients with a high suspicion of BD/DNC, precluding confirmation of BD/DNC. Consequently, only the distribution of TCD findings in this population could be described. In addition, TCD findings may have influenced decisions to initiate or continue the BD/DNC determination process in some patients, potentially introducing verification bias. Finally, the lack of concurrent validation with other cerebral blood flow assessments (e.g., angiography or nuclear medicine flow study), as well as the absence of serial neuroimaging or longitudinal TCD follow-up in some cases, limited mechanistic interpretation of persistent intracranial arterial flow after BD/DNC.

## 5 Conclusions

When TCD demonstrates cerebral circulatory arrest, the findings provide strong supportive evidence for BD/DNC. Given its advantages of being noninvasive, repeatable, and feasible at the bedside, TCD has the potential to be a useful ancillary modality for pediatric BD/DNC evaluation. However, in some children with clinically confirmed BD/DNC, TCD continues to detect intracranial arterial blood flow. Factors such as disruption of the closed intracranial system, mechanical circulatory support, cerebrovascular malformations, and timing of examination may affect the performance of TCD in confirming BD/DNC. Therefore, in cases where TCD findings are inconsistent with BD/DNC in children with a high clinical suspicion of BD/DNC or who meet clinical criteria, the findings should be interpreted in the context of these potential interfering factors and waveform characteristics. Future studies incorporating other cerebral blood flow assessment modalities are needed to further elucidate the mechanisms underlying persistent intracranial arterial flow on TCD after BD/DNC.

## Data Availability

The raw data supporting the conclusions of this article will be made available by the authors, without undue reservation.
