# Supplementary material for: Clinical utility and limitations of transcranial Doppler in evaluating pediatric brain death or death by neurologic criteria: a 10-year retrospective study
Source: Front Pediatr. 2026 Oct 20;14:1931129. doi: 10.3389/fped.2026.1931129 (PMC13727551; doi:10.3389/fped.2026.1931129)
Supplement: Supplementary file 1 [file Supplementaryfile1.docx]

Supplementary Material

# 1 Supplementary Table

**Supplementary Table 1.** Implementation and findings of ancillary tests in the BD/DNC, non-BD/DNC, and incomplete determination groups

| Number of ancillary tests performed | BD/DNC  (*n* = 47) | Non-BD/DNC  (*n* = 266) | Incomplete determination | | |
| --- | --- | --- | --- | --- | --- |
|  |  |  | Evaluation not initiated (*n* = 157) | Apnea testing not completed (*n* = 24) | Repeat examination not completed  (*n* = 18) |
| 3 ancillary tests  TCD+, EEG+, and SLSEP+  TCD+, EEG−, and SLSEP+  TCD−, EEG+, and SLSEP+ | 34 (72.3)  0 (0.0)  9 (19.1)^a^ | 0 (0.0)  0 (0.0)  0 (0.0) | 3 (1.9)  0 (0.0)  0 (0.0) | 4 (16.7)  2 (8.3)  2 (8.3)^a^ | 15 (83.3)  0 (0.0)  0 (0.0) |
| 2 ancillary tests  TCD+, EEG+  TCD+, SLSEP+  TCD+, EEG−  TCD−, EEG+  TCD−, SLSEP+  TCD−, EEG− | 3 (6.4)  1 (2.1)  0 (0.0)  0 (0.0)  0 (0.0)  0 (0.0) | 0 (0.0)  0 (0.0)  0 (0.0)  0 (0.0)  0 (0.0)  65 (24.4) | 20 (12.7)  0 (0.0)  10 (6.4)  3 (1.9)^a^  0 (0.0)  22 (14.0) | 3 (12.5)  2 (8.3)  1 (4.2)  1 (4.2)  1 (4.2)  4 (16.7) | 0 (0.0)  1 (5.6)  1 (5.6)  0 (0.0)  0 (0.0)  0 (0.0) |
| 1 ancillary test  TCD+  TCD− | 0 (0.0)  0 (0.0) | 0 (0.0)  201 (75.6) | 50 (31.8)  49 (31.2)^b^ | 4 (16.7)  0 (0.0) | 1 (5.6)  0 (0.0) |

Data are presented as *n* (%). “+” indicates findings consistent with BD/DNC. “−” indicates findings inconsistent with BD/DNC. BD, brain death; DNC, death by neurologic criteria; TCD, transcranial Doppler; EEG, electroencephalography; SLSEP, short-latency somatosensory evoked potential.

^a^One patient in each of the three combinations showed no flow signals on the initial TCD examination. ^b^Six patients showed no flow signals on the initial TCD examination.

# 2 Supplementary Figures

**Supplementary Figure 1.** TCD spectral waveforms in Case 2 on day 11 of severe disorders of consciousness. (**A**) The left middle cerebral artery demonstrates continuous forward flow with a peak systolic velocity < 50 cm/s. (**B**, **C**) The right middle cerebral artery and basilar artery demonstrate systolic spikes. Scanning parameters are labeled as follows: (a) depth; (b) gain; (c) power; (d) sample volume; (e) scale; (f) baseline; (g) audio volume; (h) envelope; and (i) probe. Mean, mean velocity; Peak, peak systolic velocity; EDV, end-diastolic velocity; PI, pulsatility index; RI, resistance index; S/D, systolic-to-diastolic ratio; HR, heart rate.

**Supplementary Figure 2.** TCD spectral waveforms in Case 1 on day 4 of severe disorders of consciousness. Reduced blood velocities are observed in the left (**A**) and right (**B**) carotid siphons, with a more pronounced reduction on the right side. Scanning parameters are labeled as follows: (a) depth; (b) gain; (c) power; (d) sample volume; (e) scale; (f) baseline; (g) audio volume; (h) envelope; and (i) probe. Mean, mean velocity; Peak, peak systolic velocity; EDV, end-diastolic velocity; PI, pulsatility index; RI, resistance index; S/D, systolic-to-diastolic ratio; HR, heart rate.

**Supplementary Figure 3.** TCD spectral waveforms in Case 5 on day 28 of severe disorders of consciousness. Reduced diastolic blood velocities and increased pulsatility index are observed in the left (**A**) and right (**B**) middle cerebral arteries. Ipsilateral common carotid artery compression resulted in a transient decrease in blood velocity (white arrow). AMPL, amplitude; Peak, peak systolic velocity; Mean, mean velocity; Dias, end-diastolic velocity; PI, pulsatility index; SD, systolic-to-diastolic ratio; HR, heart rate; LMCA, left middle cerebral artery; RMCA, right middle cerebral artery.
